# Differences in digital health literacy and future anxiety between health care and other university students in England during the COVID-19 pandemic

**DOI:** 10.1186/s12889-022-13087-y

**Published:** 2022-04-05

**Authors:** Daniel Frings, Susie Sykes, Adeola Ojo, Gillian Rowlands, Andrew Trasolini, Kevin Dadaczynski, Orkan Okan, Jane Wills

**Affiliations:** 1 School of Applied Sciences, London South Bank University, 101 London Road, London, SE1 0AA UK; 2 Institute of Health and Social Care, London South Bank University, 101 London Road, London, SE1 0AA UK; 3 Population Health Sciences Institute, Newcastle University, Newcastle upon Tyne, NE2 4BN UK; 4 Department of Nursing and Health Science, Fulda University of Applied Sciences, Leipziger Straße 123, 36037 Fulda, Germany; 5 Department of Sport and Health Sciences, Technical University Munich, Germany, Uptown München-Campus D, Georg-Brauchle-Ring 60/62, 80992 Munich, Germany

**Keywords:** COVID-19, Coronavirus, Digital health, Health information, Infodemic, Health literacy, Student, University student

## Abstract

**Background:**

This study investigates university students’ digital health literacy and web-based information-seeking behaviours during the early stages of the COVID-19 pandemic in England. It compares undergraduate and postgraduate students in non-health related subjects with health care students, many of whom were preparing for, or working in, frontline roles. The survey was conducted as part of a wider study by the COVID-HL research consortium.

**Methods:**

A cross-sectional study was conducted among *n* = 691 university students aged ≥18 years from 25 universities across England using an adapted digital survey developed by COVID-HL. Data were collected regarding sociodemographic characteristics and specific measures drawn from the Future Anxiety Scale and the Digital Health Literacy Instrument (DHLI). These had been adapted for use in an English setting and to the specific context of the COVID-19 pandemic. Other data collected included students’ anxiety or worries about the future using the Dark Future Scale as well as behaviours in online information-seeking. Data were analysed using correlations to test for relationships between constructs and also between group comparisons to test for differences between students studying health and non-health related subjects.

**Results:**

Across digital health literacy dimensions, there was no significant difference between students studying health-related subjects and other students. Health care students did report greater difficulties in relation to how to behave online. They also relied less on public body sources for information about the pandemic. A significant difference was found between the two student populations in relation to their anxiety about the future with health care students reporting fewer fears about the future.

**Conclusions:**

Although digital health literacy is well developed in university students, a significant proportion of students still face difficulties with evaluating online information which may frustrate public health efforts. This could be addressed by ensuring health students’ curriculum in particular encompasses digital health literacy.

## Background

The COVID-19 pandemic has exposed the world to a novel, highly infectious virus (severe acute respiratory syndrome coronavirus 2 (SARS-CoV-2)) to which there was no prior immunity and which causes a disease (COVID-19) with significant levels of morbidity and mortality [1]. COVID-19 was declared a Public Health Emergency of International Concern at the end of January 2020. Global deaths due to the COVID-19 pandemic were estimated by WHO to be at least 6 million by March of 2022 [2]. Bringing the pandemic under control requires population adherence to public health measures such as social distancing, hygiene and vaccination. Such adherence requires health literacy – that is‘the motivation, knowledge and competencies to access, understand, appraise and apply health information in order to make judgments and take decisions in everyday life concerning healthcare, disease prevention and health promotion to maintain or improve quality of life throughout the course of life’ [3].

A notable aspect of the COVID-19 pandemic has been the accompanying ‘infodemic’ characterized by the rapid spread and amplification of vast amounts of valid and invalid information on the internet or through other communication technologies [4]. Identifying which information is accurate and helpful requires digital health literacy or eHealth literacy, whose broad early definition is ‘the ability to seek, find, understand, and appraise health information from electronic sources and apply the knowledge gained to addressing or solving a health problem’ [5]. While digital health literacy and eHealth literacy are overlapping concepts that are often used interchangeably, eHealth literacy has a focus of information gathering from online sources and was developed in the context of Web 1.0 platforms (traditional, information-based websites, where users view content in a passive manner) and prior to Web 2.0 platforms (websites that allow user collaboration and includes social networking sites, social media sites, blogs, wikis and video sharing sites). Digital health literacy incorporates interactivity across web based platforms including social media [6]. General health literacy and digital health literacy are inter-related concepts; people at risk of lower health literacy skills are also at risk of low digital health literacy [7]. Low health literacy is a common problem; 61% of the English working age population have low health literacy, with rates of low health literacy highest amongst older people, ethnic minority groups and people living in socio-economically deprived areas [8]. Digital skills are also socio-demographically patterned [9]. Alongside facing the greatest barriers to accessing and using reliable and useful COVID-19 information these same population groups are also most at risk of higher complications and death from COVID-19 [10, 11].

A group expected to have higher health literacy and digital literacy are University students, who tend to be younger, and from less socio-economically deprived backgrounds, compared to the population as a whole [12]. Although student courses invariably demand skills in seeking and appraising information [13], the initial findings of national cross-sectional web-based surveys conducted on the levels of digital health literacy of students in Germany [14] and Portugal [15] found they have difficulties making judgements about the reliability of health information concerning COVID-19. However, little is known about the specific digital health literacy abilities of those studying to be healthcare professionals – an important group as they will be the future ‘frontline’ working with both COVID-19 and yet to be identified emerging infectious diseases.

Given their pivotal role in reducing the effects of emerging infectious diseases, healthcare professionals entering the workforce need to be prepared with new skills and competencies as they enter into an increasingly digitalised healthcare working environment. This is important as, alongside operating in such an environment, they are also required to support patients in their navigation of digital services and complex online information [16]. Despite these requirements, limited levels of digital health literacy amongst healthcare professionals have been found to be a common barrier to the implementation of digital health services [17–19]. As well as the importance of digital health literacy skills to deliver services and to support patients, Turan et al. [20], argue that satisfactory digital health literacy is important for health students’ (in this case nursing students’) own health and understanding of the importance of their personal healthy lifestyle behaviours. This is in part as a basis for their role as health promoters and to meet the expectation that they become healthy role models [21]. Thus, understanding the levels of digital health literacy amongst health students is important, in terms of guiding curricula, policy and practice. It also has a potential impact on the wellbeing of healthcare professionals, to the extent that it buffers stress and may decrease anxiety about the future.

Higher digital health literacy has been found to be associated with better health, more positive health behaviours and health knowledge [22]. In recent studies, higher levels of health literacy have been associated with less anxiety and fear about COVID-19 among medical students and may even act as a protective factor as students are better able to navigate the “infodemic” and co-existing conspiracy beliefs [23]. Not being apprehensive about the future or having negative expectations is important for the healthcare professions and in particular, for emergency preparedness. Staff with these attributes have been shown to feel less pressure and to focus on practical problems and solutions. Although most work on resiliency and self-efficacy has focused on natural disaster situations [24, 25], emerging literature from the pandemic context is consistent showing that those who are less pessimistic and reporting less fear about the future feel more psychologically prepared in pandemic management [26, 27].

Given the importance of digital health literacy in general, and for students preparing to enter health professions especially (in terms of both practice and their wellbeing), the current study aimed to explore digital health literacy levels amongst a sample of students in England. The data used to achieve this aim were collected as part of a larger multinational study undertaken by the COVID-HL research consortium [https://covid-hl.eu], a network of researchers on health literacy from more than 50 countries. Using the same instrument as the COVID-HL consortium, this study reports on the findings of students at universities in England, comparing students studying health-related disciplines who might be expected to have even higher health and digital health literacy than peers in non-health-related courses.

## Methods

A survey developed by the COVID-HL research consortium [28] was adapted, and minor amendments made to ensure questions were more applicable to the student population throughout England. The survey was divided into four sections: 1) personal information 2) life circumstances 3) information about COVID-19 4) information sources. It was written in English and included a number of question formats such as multiple choice, dropdown selections, comment boxes, rank orders and sliding scales. In total, there were 37 questions and it took 5–10 min to complete. The survey was uploaded to Qualtrics' survey management software. Each participant was allocated a participant number when starting the survey and no identifying information or names were collected.

A non-probability approach to sampling was used. The names and contact information of Faculty Deans, Associate Deans, Heads of Marketing, Deans of Health, Healthy Universities UK and the National Union of Students in universities was compiled from university websites. This group were used to disseminate the survey. An email contained details on the purpose of the study, its voluntary and confidential nature, the process involved, disadvantages and benefits to participation, what will happen with the results and the review arrangements for the study. The direct link to the survey was provided and the email contained modifiable promotional materials (email template and social media flier) for universities to use as a method to distribute to students at their corresponding school or faculty. Follow-up emails were sent to all Deans at two-week intervals for a period of 8 weeks. In addition to the email distribution, social media was used to advertise the survey to students, using matching digital social media fliers sent to universities. Snowballing was employed as a secondary sampling strategy whereby participants passed the survey link to other students.

### Participants

A total of 691 Undergraduate and Master level students were recruited from 25 universities across England (an additional 30 participants who were studying at doctoral level were also recruited but excluded from the analysis). Of these, 306 (44%) were health students. Non health students included those studying diverse disciplines including Humanities (1.4%), Languages (1.0%), Engineering (8.5%), Arts and Creative Sciences (0.3%), Earth Sciences (2.7%), Mathematics and Physics (9.8%), Economics Business (2.6%) and Social Studies (4.5%). Ages ranged from 18 to 59 years (*M *= 25.15, *SD* = 8.45). Four hundred and ninety-four (71.5%) of participants identified as female, 192 (27.8%) as male, 4 (6%) as other and 1 (0.1%) preferred not to indicate a gender. This is broadly in line with the gender distribution in UK higher education.

### Measures

#### Sociodemographic information

Participants were first asked about their gender, age and country of birth. They were then asked about their university name, the type of programme they were studying (Bachelor’s, Master’s or another type), how many semesters had they studied at university in England (current and previous degrees included). They were also asked about which group their university course came under, with “health subjects, including medicine” being listed as one of the ten options. Information about their financial situation was gathered by asking them how they financed their studies, their satisfaction with their financial situation and by using the MacArthur Scale (ten-point scale) to determine a subjective social status level [29].

### Future anxiety

The attitudes of the participants towards the future were determined by presenting a series of nine statements. The first five items were drawn from to the “Dark Future Scale” [30] a shorter version of the Future Anxiety [31] scale. The remaining five items were drawn from the Future Anxiety scale on the basis of face validity. The scale was adapted to ask participants to reflect on COVID related concerns when answering the questions (in contrast to AIDs/Cancer in the original formulation). Participants were required to rate each item on a 5-point Likert scale anchored at 1 (*decidedly false*), 2 (*probably false*), 3 (*neither true nor false*), 4 (*probably true*) and 5 (*decidedly true*). The statements were as follows: *‘I am afraid that the problems which trouble me now will continue for a long time’, ‘I am terrified by the thought that I might sometimes face life’s crises or difficulties’, ‘I am afraid that in the future my life will change for the worse’, ‘I am afraid that changes in the economic and political situation will threaten my future’, ‘I am disturbed by the thought that in the future I won’t be able to realize my goals’, ‘I fall into a state of tension and uneasiness when I think of my future affairs’, ‘I am sure that in the future I will realize the most important goals (values) in my life’, ‘I have the impression that the world tends toward collapse’, ‘I am disturbed by the possibility of a sudden accident or serious illness (e.g. cancer, COVID-19)’.* Cronbach’s α in the current study for the whole scale was 0.86. The anchors used differed from the original scale anchors, but we note the internal reliability as evidence the psychometric properties were not overly affected by this change). We refer to this scale below as the extended Dark Futures scale.

### Digital health literacy

The participants’ perceptions about their own digital health literacy were determined via a series of questions relating to how easy they found it to search for, formulate questions or add their own content, and determine the reliability and relevance of information relating to coronavirus. These questions were taken from well-established Digital Health Literacy Instrument (DHLI) [22] and adapted to the Covid-19 context (from the original focus on AIDS/Cancer). The DHLI was chosen over other tools because of its relevance to the study aim, currency and conceptual fit. The development of DHLI built on older tools such as eHEALS but include a focus on interactive digital platforms such as social media.

Four of the seven subscales from the DHLI were used (Information searching, adding self-generated content online, evaluating reliability and determining relevance). The internal consistency (Cronbach’s α) of the first four subscales was acceptable to good (0.70–0.83 in the original publication, 0.75–0.91 in the current study). To measure participants’ understanding of privacy when posting about coronavirus on social media with the following questions: ‘*Do you find it difficult to judge who can read along?’, ‘Do you (intentionally or unintentionally) share your own private information (e.g. name or address)?’, ‘Do you (intentionally or unintentionally) share some else’s private information?’* These were rated on a similar 4-point Likert type scale 1 (*often*), 2 (*several times*), 3 (*once*), 4 (*never*)*.* Subscales around operational skills and navigation skills were omitted due to an expectation that the university students would hold these more technical skills. Each of the included topics were grouped into subscales with three questions on each topic and participants were required to answer on 4-point Likert scales anchored at 1 (*very difficult*), 2 (*difficult*), 3 (*easy*), 4 (*very easy*). Due to low reliability in the original publication of the scale (Cronbach’s α = .46) and in our own presentation (Cronbach α = .51), analyses from the protecting privacy subscale are not presented in the current paper (but are available on request from the lead author).

### Sources of information

The participants were also asked to provide information about how often they used different sources to get information about coronavirus and topics relating to it, on the internet. They were asked to rate each option given on a 5-point Likert type scale anchored at 1 (*never*), 2 (*rarely*), 3 (*sometimes*), 4 (*often*), 0 (*don’t know*). The sources rated were as follows: search engines, government websites, Wikipedia (encyclopedias), health portals, social media, YouTube, blogs on health topics, guidebooks (communities), news portals and websites of doctors and health insurance companies. They also indicated which of the following languages they access information in – English, French, Spanish, German, Chinese or Other (being permitted to select multiple options).

### Analytical strategy

To account for significant skew in a number or variables (specifically, Digital Health Literacy, determining relevance and the extended Dark Future Scale and satisfaction with information online), which could not be sufficiently reduced via screening of outliers or transformations, we adopted a bootstrapping approach to analysis to allow for the use of parametric tests [32, 33]. In each instance we took 1000 bootstrapped samples, and interpret significance based on 95% confidence interval (*p* < .05). Where means are reported, lower and upper 95% confidence intervals are reported in square parentheses. Correlational analysis was used to test relationships between variables, and between subjects t tests for differences between groups.

## Results

### Relationships between variables

Descriptive statistics and relationships between key variables can be seen in Table 1. Correlational analysis showed that the extended Dark Future Scale was negatively correlated with all dimensions of Digital Health Literacy and satisfaction. Reported satisfaction with information online was positively related to all dimensions of Digital Health Literacy with the exception of the extended Dark Future Scale (where a negative relationship was observed) and the Digital Health Literacy subscale capturing confidence (in which no relationship was observed). Number of years enrolled in study did not correlate with any other measures in the whole sample analysis. Correlations between the number of years enrolled did not correlate significantly with other variables when separate analyses were undertaken for health students and non-health students (*r*s < +/− 0.09, *p*s > .05).

**Table 1:** Descriptive statistics of key variables and their zero-order correlations

	*M (SD)*	*Information search*	*Adding self-generated content*	*Evaluating reliability*	*Determining relevance*	*Satisfaction with information online*	*Number of years enrolled*
Extended Dark Future	*4.22 (2.40)*	−.21**	−.23**	−.18**	−.22**	−.25**	*.02*
Information searching	*2.95 (0.62)*	*–*	.49**	.51**	.52**	.33*	*.06*
Adding self-generated content online	*2.87 (0.64)*		*–*	.48**	55**	.19**	*.06*
Evaluating reliability of information	*2.77 (0.71)*			*–*	.62**	.27**	*.09*
Determining relevance of information	*2.94 (0.61)*				*–*	.28**	*.04*
Satisfaction with information online	*3.26 (0.86)*					–	*.02*

Note: * = p < .05, **p < .001

### Digital health literacy

As can be seen in Table 2, there were no significant differences in Digital Health Literacy between health care students and others on any of the subscales.

**Table 2:** Digital Health Literacy (DHL) amongst health and non-health students

*DHL scale*	*Health care students* *Mean [CI]*	*Non-health care students* *Mean [CIs]*	*t*	*Lower/Upper CIs*
Searching for information	2.98 [2.90,3.08]	2.92 [2.85,3.00]	1.01	−0.17,0.05
Adding self-generated content online	2.91 [2.83,3.01]	2.84 [2.76,2.91]	1.23	−0.19, 0.04
Evaluating reliability	2.80 [2.71,2.90]	2.75 [2.67,2.84]	0.81	−0.18,0.08
Determining relevance	2.95 [2.87,3.04]	2.93 [2.86,3.00]	0.40	−0.13, 0.89

*Note*: All dfs = 479. * = p = 0.049

### Sources of, and satisfaction with, information

#### Information search patterns

As can be seen in Table 3, health care students described lower use of public body websites, health blogs, health portals and websites of doctors or health insurance companies than did non-healthcare students, but there were no differences in their use of other sources.

**Table 3:** Use of information sources by health care vs. non health care students

*Source*	*Health* *Mean [CI]*	*Non-health* *Mean [CIs]*	*t*	*Lower/Upper CIs*
Search engines	1.37 [1.27, 1.48]	1.36 [1.29, 1.44]	0.45 (437}	−0.13,0.13
Websites of public bodies	1.50 [1.40, 1.60]	1.77 [1.66,1.87]	3.56 (437)	0.12, 0.41
Wikipedia and online encyclopaedias	2.80 [2.65,2.93]	2.96 [2.84,3.08]	1.67 (437)	−0.03, 0.35
Social networking sites	2.36 [2.19,2.52]	2.42 [2.28,2.55]	0.53 (437)	−0.15, 0.27
YouTube	2.60 [2.43,2.76]	2.71 [2.57,2.86]	1.05 (437)	−0.11, 0.34
Blogs on health topics	2.95 [2.81,3.09]	3.29 [3.17, 3.39]	3.74 (437)	0.16, 0.51
Guidebook communities	3.36 [3.23,3.48]	3.40 [3.29,3.48]	0.48 (379)	−0.12, 0.19
Health portals	1.69 [1.57,1.81]	2.02 [1.90,2.13]	3.89 (437)	0.16, 0.50
Websites of doctors or health insurance companies	3.02 [2.86,3.17]	3.27 [3.15,3.37]	2.63 (437)	0.63, 0.44
News portals	2.04 [1.90,2.16]	2.11 [2.00,2.23]	0.81 (437)	−0.10, 0.24

*Note dfs = 437*

#### Languages of sources

Four hundred and seventy-nine participants accessed sources in English, 10 in French, 9 in Spanish, 5 in German, 3 in Chinese and 39 in other languages.

#### Satisfaction with information

There was no difference between how satisfied health care students (*M* = 3.30 [3.18,3.42] and non-health care students (*M* = 3.24 [3.14,3.34] were in information they found online about coronavirus, *t*(479) = 0.77, CIs = − 0.22,0.95.

### Anxiety about the future

#### Dark future

Health care students scored lower (*M* = 2.36, [2.45,2.47]) on the extended Dark Future scale than did non-health care students (*M* = 2.52, [2.42,2.63]). This difference was statistically significant, *t*(479) = 2.14, CIs = 0.14,0.32.

## Discussion

All levels of healthcare provision have been impacted by a rapid development of information and communication technology over the last 30 years with digital health technology seen as a key solution to many of the current challenges facing both healthcare services and public health [34]. The urgency to prepare a digitally health literate workforce has been increased by the COVID-19 pandemic where a paradigm shift has seen increasing reliance of digital platforms and information sources, and an environment where there has been a rapid development of new information from multiple sources on the spread, prevention, treatment and policies associated with the pandemic. This ‘infodemic’ [35] of both reliable and false information has created a complex digital landscape for healthcare professionals and understanding how well-prepared healthcare students are to navigate that landscape is important.

In this study, we sought to compare levels of digital health literacy and anxiety about the future among healthcare students and non-healthcare students at UK universities immediately following the first lockdown of the COVID-19 pandemic in 2020. Our analysis shows no significant difference in levels of digital health literacy amongst those students studying for a health-related qualification and those studying a non-health related subject. There is a limited body of literature regarding levels of digital health literacy amongst health and social care students and the number of tools used to measure digital health literacy (for example, eHLQ [36]; eHLA [37]; eHEALs [38]), which makes it hard to compare studies directly. However, digital health literacy skills do not seem to be more fully developed amongst the future healthcare workforce than amongst their student peers. This set of findings is reflected in some but not all of the wider literature on digital health literacy amongst healthcare students in a non-pandemic context. For instance, while a Danish study found levels of digital health literacy to be satisfactory [16] and a further Danish study found that digital health literacy levels among healthcare students were higher than that of the general population [39], a study of European medical students found that 53.2% self-reported their digital health literacy skills as poor or very poor [17]. Other studies with student nurses have shown that undergraduate nursing students may be aware of the available online health resources and be able to access them but that they face challenges in appraising information and differentiating between high- and low-quality sources [40, 41] and that they face difficulties in using information to make decisions [42].

While no other studies have been found that specifically make the comparison between the digital health literacy skills of healthcare and non-healthcare students, some studies have been conducted to compare skills associated with the broader but related concept of health literacy. Findings from these suggest that health literacy levels among health students are typically higher than those of other student groups [43, 44], in contrast to this study. A further variation in findings is that while the existing literature shows digital health literacy increasing as students progress through academic years [16, 20, 40, 41, 45, 46], our study found digital health literacy levels remained the same throughout the academic year groups for both healthcare and non-healthcare students. Caution must be exercised directly comparing these studies, as they used a variety of differing scales. This nonetheless prompts a consideration of whether these differences may be associated with the particular pandemic context within which this study was undertaken or that the educational curriculum offered to these students in England does not prioritise or address the use of digital information.

The uniqueness of the COVID-19 context is important. Health literacy and its related concepts such as digital health literacy have been described as context specific with individual health literacy varying according to the context within which people are called on to apply it [47–50]. Data were collected 3 months after the first COVID-19 related lockdown in England, a time of unprecedented volume and pace of information about a new health condition that was impacting on the whole population on an emergency basis. Digital health literacy has been described as a process-oriented skill set that evolves over time as new technologies are introduced and which need to respond to the changing contexts at a personal, social and environmental level [50]. The pace of change at all of these three levels during the early stages of the pandemic may have impacted on digital health literacy skills.

It is however a concern that health students in England, even in their final year of study and preparing to enter the workforce, are not better equipped to manage this information than their peers from other academic disciplines. Our findings show that not only are their digital health literacy levels no higher than non-health students, but they are less likely to search for health information from public body websites, health blogs, health portals and websites of doctors than non -health care students. These are sources that students on a health-related course might reasonably be expected to have been introduced to through their studies. Internationally, there are frequent calls in the literature for a fuller integration of digital health literacy skills into the medical and nursing curricula [34, 51] as well as calls from medical students themselves for all aspects of eHealth to be more fully integrated into the curriculum [34] showing a desire to act as digital health literacy mediators for patients and for digital health literacy to be a prerequisite competency for healthcare professionals [34, 51]. Responses to these calls have varied internationally. In England, Health Education England and the Royal College of Nursing in the UK published a framework outlining commitments to improve the digital literacy education of the health and social care workforce [52] but the focus of this has been on developing skills in the use of digital technology rather than on health literacy skills in an online environment. The findings from this study suggest that in addition to the research and evidence digital skills developed amongst university students, there should also be attention to the skills needed to navigate an online environment of information. The pandemic exposed the amount of misinformation and variety of information sources about health and placed the skills to critically evaluate health claims at a premium. This study also reflects previous findings about the importance of healthcare students having education about safe and professional behaviour online [53].

Our findings go on to show that while health care students did not show greater levels of digital health literacy than their counterparts from other subjects, they did show less anxiety about the future. The reasons for these lower levels of anxiety amongst health care students in England (who as a group do not have higher levels of digital health literacy) are not clear but the context of the pandemic should be considered. There may have been an enthusiasm to be part of the frontline –evidenced in rising numbers of applications to become nursing students with numbers accepted onto nursing courses rising by 23.8% between 2019 and 2020 and those accepted to study medicine rising by more than a third since 2017 [54]. While changes in financial arrangements for students may have contributed to this, the Chief Nursing Officer for Nursing and the Royal College of Nursing have attributed it largely to the pandemic, highlighting the increased attention given to the importance of the role played by nurses and a new respect for their work [55]. There seems to be an association between the “nurse as hero” discourse during the pandemic, levels of digital health literacy and future anxiety. The enthusiasm to be part of the health care professions exhibited during the pandemic may override the motivation shown by other students to engage with the huge slew of information and their levels of apprehension and may account for the lower levels of access to information by health care students.

### Limitations

The measure of digital health literacy is based on self-reported status rather than an objective measure which may therefore introduce a bias. The sample may also not necessarily be representative of all university students and particularly of health and social care students across England. University students are more highly educated than other parts of the population limiting the transferability of findings to other parts of the population. All data were collected using online mechanisms potentially excluding any students that did not have access to the internet. However, as most university courses require some form of online engagement it is not anticipated that this reflects a serious limitation.

## Conclusion

This study adds to an emerging body of evidence regarding the ways in which population groups have engaged with information about the COVID-19 pandemic. University students might be expected to have higher levels of both health literacy and digital health literacy and yet several studies have shown that they also have struggled with the volume and reliability of available information online [14] and that universities have a role in expanding digital learning [56, 57] especially as students are active agents of information.

This study shows that healthcare students in England had no greater levels of digital health literacy during the pandemic than other students, despite their study discipline and some choosing to work in the frontline; whilst a study of medical students found that health literacy was protective during the pandemic [23], this study did not find an association between the ability to access, analyse and apply online health information with fear of COVID-19. Yet the healthcare students in this study did show lower levels of fear illustrating the importance of understanding health literacy and digital health literacy as a relational concept in which individuals interact with context and the “nurse as hero’ discourse that has found expression at all levels of society and may have acted as a counterweight to the “infodemic” [58].

## Data Availability

Upon reasonable request, data will be made available via the Covid-HL Network https://covid-hl.eu

## Abbreviations

DHL: Digital Health Literacy
DHLI: Digital Health Literacy Instrument

## References

[1] ECDC (2020). Communicable disease threats report.

[2] World Health Organisation (2022). WHO COVID-19 dashboard.

[3] Sørensen K, Van Den Broucke S, Fullam J, Doyle G, Pelikan J, Slonska Z (2012). Health literacy and public health: a systematic review and integration of definitions and models. BMC Public Health.

[4] Hua J, Rajib S. Corona virus (COVID-19) “Infodemic” and emerging issues through a data lens: the case of China. Int J Environ Res Public Health. 2020;17 Available from: 10.1016/j.ijid.2020.04.033%0Ahttp://www.ncbi.nlm.nih.gov/pubmed/32460566. 10.3390/ijerph17072309 PMC7177854 32235433

[5] Norman CD, Skinner HA, Norman CD, Street E (2006). eHealth Literacy : essential skills for consumer health in a networked world. J Med Internet Res.

[6] van der Vaart R, Drossaert C (2017). Development of the digital health literacy instrument: measuring a broad Spectrum of health 1.0 and health 2.0 skills. J Med Internet Res.

[7] Estacio EV, Whittle R, Protheroe J. The digital divide: examining socio-demographic factors associated with health literacy, access and use of internet to seek health information. J Health Psychol. 2017:135910531769542 [cited 2019 Jan 10]. Available from: http://journals.sagepub.com/doi/10.1177/1359105317695429. 10.1177/1359105317695429 28810415

[8] Rowlands G, Protheroe J, Winkley J, Richardson M, Seed PT, Rudd R (2015). A mismatch between population health literacy and the complexity of health information: an observational study. Br J Gen Pract.

[9] Hurst DD (2021). Mind the gap: the digital divide and digital inclusion.

[10] Marmot M, Allen J, Goldblatt P, Herd E, Morrison J. Build Back fairer: the Covid-19 Marmot review. Heal Found. 2020; Available from: https://www.health.org.uk/publications/build-back-fairer-the-covid-19-marmot-review.

[11] Public Health England. Disparities in the risk and outcomes of COVID-19. PHE Publ. 2020;89 Available from: https://www.gov.uk/government/publications/covid-19-review-of-disparities-in-risks-and-outcomes.

[12] HESA Higher Education Student Statistics. Higher Education Student Statistics: UK, 2017/18 - Student numbers and characteristics. High Educ Student Stat UK, 2017/18 - Student numbers Charact | HESA. 2020;1–5. Available from: https://www.hesa.ac.uk/news/17-01-2019/sb252-higher-education-student-statistics/numbers

[13] Stellefson M, Hanik B, Chaney B, Chaney D, Tennant B, Chavarria EA (2011). eHealth literacy among college students: a systematic review with implications for eHealth education. J Med Internet Res.

[14] Dadaczynski K, Okan O, Messer M, Leung AYM, Rosário R, Darlington E (2021). Digital health literacy and web-based information-seeking behaviors of university students in Germany during the COVID-19 pandemic: cross-sectional survey study. J Med Internet Res.

[15] Rosário R, Martins MRO, Augusto C, Silva MJ, Martins S, Duarte A (2020). Associations between covid-19-related digital health literacy and online information-seeking behavior among portuguese university students. Int J Environ Res Public Health.

[16] Holt KA, Overgaard D, Engel LV, Kayser L (2020). Health literacy, digital literacy and eHealth literacy in Danish nursing students at entry and graduate level: a cross sectional study. BMC Nurs.

[17] Machleid F, Kaczmarczyk R, Johann D, Balčiūnas J, Atienza-Carbonell B, Von Maltzahn F, et al. Digital Health in Medical Education: Findings from a Mixed-Methods Survey among European Medical Students. 2020; 10.2196/preprints.19827. 10.2196/19827 PMC7455864 32667899

[18] Schreiweis B, Pobiruchin M, Strotbaum V, Suleder J, Wiesner M, Bergh B (2019). Barriers and facilitators to the implementation of eHealth services: systematic literature analysis. J Med Internet Res.

[19] National Health Service (2018). Digital literacy capability framework (United Kingdom).

[20] Turan N, Güven Özdemir N, Çulha Y, Özdemir Aydın G, Kaya H, Aştı T (2021). The effect of undergraduate nursing students’ e-health literacy on healthy lifestyle behaviour. Glob Health Promot.

[21] Kelly M, Wills J, Sykes S. Do nurses’ personal health behaviours impact on their health promotion practice? A systematic review. Int J Nurs Stud. 2017;76:62–77. 10.1016/j.ijnurstu.2017.08.008 28938104

[22] Van der Vaart R, Drossaert C (2017). Development of the digital health literacy Instrument : measuring a broad Spectrum of health.

[23] Nguyen HT, Do BN, Pham KM, Kim GB, Dam HTB, Nguyen TT (2020). Fear of COVID-19 scale—associations of its scores with health literacy and health-related behaviors among medical students. Int J Environ Res Public Health.

[24] Boldor N, Bar-Dayan Y, Rosenbloom T, Shemer J, Bar-Dayan Y (2012). Optimism of health care workers during a disaster: a review of the literature. Emerg Health Threats J.

[25] Luthans KW, Lebsack SA, Lebsack RR (2008). Positivity in healthcare: relation of optimism to performance. J Health Organ Manag.

[26] Gandhi S, Sahu M, Radhakrishnan G, Nattala P, Sudhir P, Balachandran R. Psychological preparedness for pandemic (COVID-19) management: perceptions of nurses and nursing studetns in India. PLoS ONE. 2021:16(8):e0255772. 10.1371/journal.pone.0255772 PMC8362956 34388177

[27] Duplaga M, Grysztar M (2021). The association between future anxiety, health literacy and the perception of the COVID-19 pandemic: a cross-sectional study. Healthcare (Basel, Switzerland).

[28] Dadaczynski K, Okan O, Messer M, Leung AYM, Rosário R, Darlington E (2021). Digital health literacy and online information seeking in times of COVID-19. A cross-sectional survey among university students in Germany. J Med Internet Res.

[29] Adler NE, Epel ES, Castellazzo G, Ickovics JR (2000). Relationship of subjective and objective social status with psychological and physiological functioning: preliminary data in healthy. Health Psychol.

[30] Zaleski Z, Sobol-Kwapinska M, Przepiorka A, Meisner M (2019). Development and validation of the dark future scale. Time Soc.

[31] Zaleski Z (1996). Future anxiety: concept, measurement, and preliminary research. Personal Individ Differ.

[32] Efron B (1979). Bootstrap methods: another look at the jackknife. Ann Stat.

[33] Sainani KL (2012). Dealing with non-normal data. PM&R..

[34] Machleid F, Kaczmarczyk R, Johann D, Baleiùnas J, Atienza-Carbonell B, von Maltzahn F (2020). Perceptions of digital health education among European medical students: mixed methods survey. J Med Internet Res.

[35] Zarocostas J (2020). How to fight an infodemic. Lancet (London, England).

[36] Kayser L, Kushniruk A, Osborne RH, Norgaard O, Turner P (2015). Enhancing the effectiveness of consumer-focused health information technology systems through eHealth literacy: a framework for understanding users’ needs. JMIR Hum Factors.

[37] Furstrand D, Kayser L. Development of the eHealth literacy assessment toolkit, eHLA. Stud Health Technol Infom. 2015;216:971. 26262273

[38] Norman CD, Skinner HA. eHEALS: the eHealth literacy scale. J Med Internet Res. 2006;8(4):e27. 10.2196/jmir.8.4.e27 PMC1794004 17213046

[39] Elsborg L, Krossdal F, Kayser L (2017). Health literacy among Danish university students enrolled in health-related study programmes. Scand J Public Health.

[40] Tubaishat A, Habiballah L (2016). eHealth literacy among undergraduate nursing students. Nurse Educ Today.

[41] Park H, Lee E (2015). Self-reported eHealth literacy among undergraduate nursing students in South Korea: a pilot study. Nurse Educ Today.

[42] Rathnayake S, Senevirathna A (2019). Self-reported eHealth literacy skills among nursing students in Sri Lanka: a cross-sectional study. Nurse Educ Today.

[43] Lestari P, Handiyani H (2017). The higher level of health literacy among health students compared with non-health students. UI Proc Heal Med.

[44] Joseph R, Fernandes S, Hyers L, O’Brien K (2016). Health literacy: a cross-disciplinary study in American undergraduate college students. J Inf Lit.

[45] Balmer D, King A, Moloney W, Moselen E, Dixon R (2020). Nursing students and health literacy: the effect of region and programme level. Nurse Educ Pract.

[46] Adil A, Usman A, Khan NM, Mirza FI (2021). Adolescent health literacy: factors effecting usage and expertise of digital health literacy among universities students in Pakistan. BMC Public Health.

[47] Levin-Zamir D, Bertschi I (2018). Media health literacy, Ehealth literacy, and the role of the social environment in context. Int J Environ Res Public Health.

[48] Abel T, Hofmann K, Ackermann S, Bucher S, Sakarya S (2015). Health literacy among young adults: a short survey tool for public health and health promotion research. Health Promot Int.

[49] Abel T, McQueen D (2021). Critical health literacy in pandemics: the special case of COVID-19. Health Promot Int.

[50] Brørs G, Norman CD, Norekvål TM (2020). Accelerated importance of eHealth literacy in the COVID-19 outbreak and beyond. Eur J Cardiovasc Nurs.

[51] Jimenez G, Spinazze P, Matchar D, Koh Choon Huat G, van der Kleij RMJJ, Chavannes NH (2020). Digital health competencies for primary healthcare professionals: a scoping review. Int J Med Inform.

[52] Cumming I, Davies J. Health education England. Improving Digital Literacy. 2017;11 Available from: https://www.rcn.org.uk/professional-development/publications/pub-006129.

[53] Ramage C, Moorley C (2019). A narrative synthesis on healthcare students use and understanding of social media: implications for practice. Nurse Educ Today.

[54] UCAS (2020). What happened to the Covid Cohort ?.

[55] Ford M. Latest data confirms ‘record’ rise in nursing students for 2020. Nursing Times. 2020. Available from https://www.nursingtimes.net/news/education/latest-data-confirms-record-rise-in-nursing-students-for-2020-16-12-2020/. Accessed Dec 2020.

[56] Patil U, Kostareva U, Hadley M, Manganello JA, Okan O, Dadaczynski K (2021). Health literacy, digital health literacy, and COVID-19 pandemic attitudes and behaviors in U.S. college students: implications for interventions. Int J Environ Res Public Health.

[57] Zakar R, Iqbal S, Zakar MZ, Fischer F (2021). COVID-19 and health information seeking behavior: digital health literacy survey amongst university students in Pakistan. Int J Environ Res Public Health.

[58] Mohammed S, Peter E, Killackey T, Maciver J (2021). The ‘nurse as hero’ discourse in the COVID-19 pandemic: a poststructural discourse analysis. Int J Nurs Stud.

